# Mortality of Philadelphia for January, February and March, 1853, Arranged from the Record Kept at the Health Office

**Published:** 1853-05

**Authors:** Wilson Jewell


*Mortality of Philadelphia for January, February and March, 1853, arranged from the Record kept at the Health Office.* By WILSON JEWELL, M. D.

The total number of deaths from all causes for the first quarter of the present year, beginning Jan. 1st and ending April 2d, including a period of 92 days, or 13 weeks and one day, amounts to 2478, averaging 27 deaths per day.

By deducting the "Still Born," the deaths from "External Causes, Old Age, Debility and Unknown," we have left 2033 deaths from known diseases.

The excess of deaths in the sexes is on the side of the males, amounting to 138.

The male still-born children almost double in number those of the female sex.

Eight hundred and seventy-five, or 36 per cent. of the deaths from all causes, exclusive of Still-Born, were in children under five years of age. Of these, the diseases of the organs of Respiration, Convulsions and Croup, make up two fifths of the whole.

Nearly 20 per cent. of all the deaths, took place in individuals under 19 years of age.

The aggregate of deaths fall short of those for the same months in 1852 by 12½ per cent. This difference is about equivalent to the decrease in the mortality from Small Pox, as presented in the two tables. The 1st quarter of 1852 showed 258 deaths from Small Pox; the present year, for the like period, gives only 29.

CLASS I. Among the deaths classified as Endemic or Zymotic, there is a decrease of 20 per cent. from those for the same months and in the same class in 1852, while the deaths from Croup, Scarlet, Typhus and Typhoid Fevers have increased over 40 per cent. The discrepancy, however, may be reconciled by the great falling off in the deaths from Small Pox. Scarlet Fever provides 223 deaths in this class, in proportion, nearly, as one to two of all others under the same head. The disease was most fatal in February, and the period of life when the greatest mortality occurred was between 2 and 5 years.

Class II. Of the Sporadics, or diseases of Uncertain Seat, there were 315 deaths. Ninety-seven, or nearly one-third, are charged to that exceedingly indefinite cause of death, Debility; and of these 42, or nearly one-half, were within the first year of life.

Class III. Under the head of Nervous Diseases, we find Convulsions occupying a very prominent place in the scale. Out of 404 deaths, they are made to furnish 133! sixty-nine of these were under one year of age.

Class IV. The deaths from diseases of the Organs of Respiration amount to 596, exceeding those of any other class, and are as one to three and a half of the whole number. Compared with those of the same class and period for last year, there is a falling off of 20 per cent.

Consumption of the Lungs holds a very conspicuous position in this table, furnishing 344 deaths, only 14 less than for the same quarter of 1852, notwithstanding the extreme mildness of the season. This fact appears to sustain the opinion, that neither temperature nor seasons afford any great exemption from its uniform fatality. The deaths recorded from Consumption of the Lungs, occupy thirteen distinct periods of life, but observe no equality. The particular age during which it seems to have been more frequently fatal was between 20 and 30, when one-third of the whole number recorded occurred. This peculiarity corresponds with other statistics. Among females the deaths were 15 per cent. more than in males.

Pneumonia stands next highest, namely, 113. A large proportion of these—more than one-half—took place within the fifth year of life. Children would seem to be its victims in a peculiar degree. Compared with those for '52 in the same quarter there is a falling off of 42 per cent.

Class VIII. The diseases of the Organs of Generation supply only 34 deaths. Of this number 32 were females, and 16, or one-half, are ascribed to Child Bed and Puerperal Fever. If we base the calculation upon 2800 births quarterly, it will give us one death in every 175 deliveries. During the same period in 1852 there were 27 deaths recorded from Puerperal Fever and one from Child Bed.

The table marked No. 3 gives a summary of the number of deaths during the quarter, at fifteen distinct periods of life. From this it appears that 470, or 18 per cent., took place within the first year of life, and 1036, or 42 per cent., were under five years. These calculations include the Still-Born. Beyond 70 years there were 141 deaths, or 6 per cent. of the whole number recorded. There were 12 deaths over 90, and one over 100 years of age.

TABLE No. I.

*Deaths for the First Quarter of 1853, classified.*

| | January. | February. | March. | Male. J. | Male. F. | Male. M. | Female. J. | Female. F. | Female. M. | Total. |
|---|---|---|---|---|---|---|---|---|---|---|
| 1. *Endemic and Contagious Diseases.* Zymotic or Epidemic | 164 | 193 | 183 | 107 | 103 | 90 | 57 | 90 | 93 | 540 |
| 2. *Uncertain or general seat.* Sporadic diseases | 89 | 101 | 125 | 41 | 56 | 68 | 48 | 45 | 57 | 315 |
| 3. Nervous System | 123 | 134 | 147 | 59 | 82 | 77 | 64 | 52 | 70 | 404 |
| 4. Organs of Respiration | 195 | 153 | 248 | 100 | 75 | 114 | 95 | 78 | 134 | 596 |
| 5. " Circulation | 17 | 14 | 25 | 7 | 12 | 12 | 10 | 2 | 13 | 56 |
| 6. Digestive Organs | 45 | 48 | 53 | 31 | 22 | 22 | 14 | 26 | 31 | 146 |
| 7. Urinary Organs | 2 | 5 | 7 | 1 | 3 | 6 | 1 | 2 | 1 | 14 |
| 8. Organs of Generation | 8 | 13 | 13 | | | 2 | 8 | 13 | 11 | 34 |
| 9. " Locomotion | 4 | 6 | 9 | 1 | 2 | 5 | 3 | 4 | 4 | 19 |
| 10. Integumentary System | | 1 | 5 | | | 3 | | 1 | 2 | 6 |
| 11. Old Age | 12 | 15 | 12 | 5 | 5 | 5 | 7 | 10 | 7 | 39 |
| 12. External Causes | 29 | 30 | 38 | 18 | 14 | 22 | 11 | 6 | 16 | 87 |
| Still Born | 52 | 58 | 51 | 34 | 36 | 32 | 18 | 22 | 19 | 161 |
| Unknown | 18 | 15 | 28 | 9 | 9 | 18 | 9 | 6 | 10 | 61 |
| | 758 | 776 | 944 | 413 | 419 | 476 | 345 | 357 | 468 | 2478 |

### TABLE No. II.

### 1. *Endemic and Contagious Diseases.—Zymotic or Epidemic.*

| | Male. | Female. | Under 1 yr. | 1 to 2. | 2 to 5. | 5 to 10. | 10 to 15. | 15 to 20. | 20 to 30. | 30 to 40. | 40 to 50. | 50 to 60. | 60 to 70. | 70 to 80. | 80 to 90. | 90 to 100. | 100 to 110. | Total. |
|---|---|---|---|---|---|---|---|---|---|---|---|---|---|---|---|---|---|---|
| Cholera Infantum | 2 | 3 | 5 | | | | | | | | | | | | | | | 5 |
| Croup | 59 | 50 | 15 | 27 | 52 | 15 | | | | | | | | | | | | 109 |
| Diarrhœa | 6 | 3 | 3 | | 1 | 1 | | | | 2 | | | | 2 | | | | 9 |
| Dysentery | 14 | 6 | 4 | 3 | 2 | 1 | | 1 | 1 | 1 | 2 | 2 | 2 | 1 | | | | 20 |
| Erysipelas | 13 | 17 | 12 | 4 | 1 | | | 2 | 2 | 3 | | 3 | 2 | | 1 | | | 30 |
| Fever | 1 | | | | 1 | | | | | | | | | | | | | 1 |
| " Bilious | 2 | 4 | | 1 | | 1 | | 2 | 1 | | | | | | 1 | | | 6 |
| " Congestive | 2 | | | | | 2 | | | | | | | | | | | | 2 |
| " Remittent | 1 | 1 | | | | 1 | | | | | | | | 1 | | | | 2 |
| " Scarlet | 124 | 99 | 20 | 42 | 112 | 40 | 5 | 1 | 2 | | | | 1 | | | | | 223 |
| " Typhus | 35 | 15 | | | | 1 | | 5 | 15 | 12 | 10 | 4 | 2 | 1 | | | | 50 |
| " Typhoid | 24 | 19 | | 2 | 1 | 3 | 1 | 2 | 6 | 9 | 9 | 6 | 3 | 1 | | | | 43 |
| Hooping Cough | 1 | 2 | 2 | 1 | | | | | | | | | | | | | | 3 |
| Measles | 4 | 2 | | 4 | 2 | | | | | | | | | | | | | 6 |
| Small Pox | 11 | 18 | 11 | 5 | 5 | 3 | 1 | 2 | | 2 | | | | | | | | 29 |
| Influenza | 1 | 1 | | | | | | | | 1 | | | 1 | | | | | 2 |
| | 300 | 240 | 72 | 89 | 177 | 68 | 7 | 15 | 27 | 30 | 21 | 15 | 11 | 6 | 2 | | | 540 |

### 2. *Uncertain or General Seat.—Sporadic Diseases.*

| | Male. | Female. | Under 1 yr. | 1 to 2. | 2 to 5. | 5 to 10. | 10 to 15. | 15 to 20. | 20 to 30. | 30 to 40. | 40 to 50. | 50 to 60. | 60 to 70. | 70 to 80. | 80 to 90. | 90 to 100. | 100 to 110. | Total. |
|---|---|---|---|---|---|---|---|---|---|---|---|---|---|---|---|---|---|---|
| Abscess | 2 | | | | | 1 | 1 | | | | | | | | | | | 2 |
| " Neck | 2 | | 1 | | | | | | 1 | | | | | | | | | 2 |
| " Throat | | 1 | | | 1 | | | | | | | | | | | | | 1 |
| Angina | 2 | 1 | | | 2 | | | | | 1 | | | | | | | | 3 |
| Cancer | 5 | 3 | | | | | | | | 2 | 1 | 1 | 4 | | | | | 8 |
| " Breast | | 2 | | | | | | | | | | | 1 | 1 | | | | 2 |
| Cyanosis | 1 | 1 | 1 | | 1 | | | | | | | | | | | | | 2 |
| Debility | 42 | 55 | 42 | 2 | | 1 | | 3 | 2 | 6 | 7 | 4 | 14 | 12 | 1 | 3 | | 97 |
| Disease of Throat | 2 | | 1 | | 1 | | | | | | | | | | | | | 2 |
| Dropsy | 27 | 24 | 1 | 1 | 10 | 6 | 1 | 2 | 5 | 2 | 2 | 5 | 11 | 4 | 1 | | | 51 |
| Enlarg't of Parotid Glands | 1 | | | | | | | | | 1 | | | | | | | | 1 |
| Hectic Fever | 3 | 1 | | 1 | | | | | 1 | 1 | | 1 | | | | | | 4 |
| Hemorrhage | 3 | 2 | 1 | | | | 1 | | 1 | 2 | | | | | | | | 5 |
| " Umbilicus | 1 | 1 | 2 | | | | | | | | | | | | | | | 2 |
| nanition | 10 | 4 | 10 | 1 | | | | | | 1 | | 1 | 1 | | | | | 14 |
| nflammation | 1 | 3 | 1 | 1 | | | | | 1 | | | | | | | 1 | | 4 |
| " Throat | 1 | 2 | 1 | | 1 | | | | | 1 | | | | | | | | 3 |
| " Parotid Glands | | 3 | 2 | | | | | | 1 | | | | | | | | | 3 |
| Malformation | 8 | 1 | 9 | | | | | | | | | | | | | | | 9 |
| Marasmus | 34 | 26 | 39 | 9 | 5 | | 1 | 1 | | 1 | | 1 | 2 | 1 | | | | 60 |
| Mortification | 1 | | | | | | | | | | | | | 1 | | | | 1 |
| " of Neck | 1 | | | | | | | | | | | 1 | | | | | | 1 |
| Scrofula | 8 | 11 | 4 | 4 | 4 | 1 | 3 | 1 | 1 | | 1 | | | | | | | 19 |
| Scirrhus | 2 | | | | | | | | 1 | | | | | | 1 | | | 2 |
| Tabes Mesenterica | 4 | 5 | 4 | 1 | 2 | | 1 | | 1 | | | | | | | | | 9 |
| Tumors | | 1 | | | | | | | | 1 | | | | | | | | 1 |
| " of Abdomen | | 1 | | | | | | | | | | | 1 | | | | | 1 |
| Ulceration | | 1 | | | | | | | 1 | | | | | | | | | 1 |
| " of Throat | 4 | 1 | | | 2 | 1 | | | 1 | | | 1 | | | | | | 5 |
| | 165 | 150 | 119 | 20 | 29 | 10 | 8 | 7 | 17 | 19 | 11 | 15 | 34 | 19 | 3 | 4 | | 315 |

3. *Of the Nervous System.*

| | Male. | Female. | Under 1 yr. | 1 to 2. | 2 to 5. | 5 to 10. | 10 to 15. | 15 to 20. | 20 to 30. | 30 to 40. | 40 to 50. | 50 to 60. | 60 to 70. | 70 to 80. | 80 to 90. | 90 to 100. | 100 to 110. | Total. |
|---|---|---|---|---|---|---|---|---|---|---|---|---|---|---|---|---|---|---|
| Apoplexy . . . | 12 | 18 | 2 | | 1 | | | | 3 | 3 | 3 | 7 | 4 | 3 | 4 | | | 30 |
| Concussion of Brain . | | 1 | | | | | | | 1 | | | | | | | | | 1 |
| Congestion " . | 16 | 20 | 9 | 5 | 8 | 3 | | | 2 | 3 | 5 | | | 1 | | | | 36 |
| Compression " . | 1 | 1 | | | | | | | | 1 | | | 1 | | | | | 2 |
| Coma . . . | 1 | | | | | | | | | | 1 | | | | | | | 1 |
| Convulsions . . | 76 | 57 | 69 | 23 | 24 | 5 | | 4 | 3 | 3 | | 1 | 1 | | | | | 133 |
| " Puerperal . | | 1 | | | | | | | 1 | | | | | | | | | 1 |
| Cramp . . . | 1 | 1 | 1 | | 1 | | | | | | | | | | | | | 2 |
| Disease of Brain . . | 9 | 12 | 9 | 3 | 3 | 2 | 1 | | 1 | 1 | | | | 1 | | | | 21 |
| Dropsy " . . | 32 | 19 | 24 | 16 | 9 | 1 | | 1 | | | | | | | | | | 51 |
| Effusion " . . | 18 | 18 | 5 | 8 | 8 | 3 | | 2 | 2 | 3 | 1 | 1 | 2 | 1 | | | | 36 |
| Epilepsy . . . | | 2 | | | 1 | | | | 1 | | | | | | | | | 2 |
| Inflammation of Brain . | 28 | 18 | 11 | 9 | 12 | 6 | 1 | | 4 | 1 | 2 | | | | | | | 46 |
| Mania . . . | 1 | | | | | | | | | 1 | | | | | | | | 1 |
| " a potu . . | 9 | 5 | | | | | | | 3 | 5 | 3 | 2 | | 1 | | | | 14 |
| Palsy . . . . | 9 | 8 | 1 | | | 1 | | | 1 | 3 | 3 | 1 | 1 | 1 | 5 | | | 17 |
| Softening of Brain . | 3 | 3 | 1 | | | | | | 3 | | | 2 | | | | | | 6 |
| Tetanus . . . | 2 | 2 | 2 | | | 1 | 1 | | | | | | | | | | | 4 |
| | 218 | 186 | 134 | 61 | 67 | 22 | 3 | 7 | 25 | 24 | 18 | 14 | 9 | 8 | 9 | | | 404 |

4. *Organs of Respiration.*

| | Male. | Female. | Under 1 yr. | 1 to 2. | 2 to 5. | 5 to 10. | 10 to 15. | 15 to 20. | 20 to 30. | 30 to 40. | 40 to 50. | 50 to 60. | 60 to 70. | 70 to 80. | 80 to 90. | 90 to 100. | 100 to 110. | Total. |
|---|---|---|---|---|---|---|---|---|---|---|---|---|---|---|---|---|---|---|
| Abscess of Lungs . | 2 | 1 | 1 | | | | | | | | | 1 | | 1 | | | | 3 |
| " Thorax . | 1 | | | | | | | | | 1 | | | | | | | | 1 |
| Asthma . . . | 2 | 2 | | | | | | | | | | 1 | 1 | 2 | | | | 4 |
| Congestion of Lungs . | 6 | 11 | 6 | 1 | 2 | 1 | 1 | | | | 1 | 1 | 1 | 2 | 1 | | | 17 |
| Consumption " . | 158 | 186 | 3 | 6 | 6 | 8 | 4 | 27 | 116 | 76 | 49 | 29 | 12 | 6 | 2 | | | 344 |
| Disease of Lungs . | 3 | 2 | 2 | | | | | 1 | | 1 | | | 1 | | | | | 5 |
| " Chest . . | 1 | | 1 | | | | | | | | | | | | | | | 1 |
| Dropsy of Chest . . | 9 | 3 | | | 2 | 1 | | | 2 | 2 | | 3 | 2 | | | | | 12 |
| Effusion " . . | | 3 | | | | 1 | | | 1 | | | | | 1 | | | | 3 |
| Hemorrhage of Lungs . | 3 | 2 | | | | | | | 3 | | | 2 | | | | | | 5 |
| Inflammation of Bronchiæ | 28 | 39 | 27 | 9 | 13 | 5 | 1 | 1 | 2 | 2 | | 1 | 3 | 1 | 2 | | | 67 |
| " Chest . | 3 | 1 | 2 | | 2 | | | | | | | | | | | | | 4 |
| " Larynx . | 4 | 5 | 1 | 1 | 3 | 1 | | | 1 | | 1 | | 1 | | | | | 9 |
| " Lungs . | 63 | 50 | 33 | 17 | 14 | 10 | | 4 | 5 | 7 | 4 | 5 | 4 | 8 | 1 | 1 | | 113 |
| " Pleura . | 3 | | | | | | | | 1 | | | 1 | 1 | | | | | 3 |
| " Tonsils . | 1 | 1 | | 1 | 1 | | | | | | | | | | | | | 2 |
| Tuberculosis . . | 2 | 1 | | | 1 | | | | | 1 | | | | 1 | | | | 3 |
| | 289 | 307 | 76 | 35 | 44 | 27 | 6 | 33 | 131 | 90 | 55 | 44 | 26 | 22 | 6 | 1 | | 596 |

### 5. *Organs of Circulation.*

| | Male. | Female. | Under 1 yr. | 1 to 2. | 2 to 5. | 5 to 10. | 10 to 15. | 15 to 20. | 20 to 30. | 30 to 40. | 40 to 50. | 50 to 60. | 60 to 70. | 70 to 80. | 80 to 90. | 90 to 100. | 100 to 110. | Total. |
|---|---|---|---|---|---|---|---|---|---|---|---|---|---|---|---|---|---|---|
| Anæmia . . . | 3 | 2 | | | | | 1 | | 1 | | 1 | | | 2 | | | | 5 |
| Aneurism Carotid . | 1 | | 1 | | | | | | | | | | | | | | | 1 |
| Congestion of Heart . | | 1 | | | | | | | | | | | | | 1 | | | 1 |
| Disease of Heart . . | 17 | 13 | 3 | | 2 | 1 | | 1 | 2 | 1 | 7 | 4 | 8 | 1 | | | | 30 |
| Dropsy " . . | 1 | 3 | | 1 | 1 | | 1 | | 1 | | | | | | | | | 4 |
| Enlargement of Heart . | 3 | 2 | | | | 1 | 2 | | | | 1 | | | 1 | | | | 5 |
| Inflammation " . | 3 | 3 | | 1 | | | 1 | | 1 | 1 | | 2 | | | | | | 6 |
| " Vein . | | 1 | | | | 1 | | | | | | | | | | | | 1 |
| Malformation of Heart . | 1 | | | 1 | | | | | | | | | | | | | | 1 |
| Ossification " . | 1 | | | | | | | | | | | | 1 | | | | | 1 |
| Syncope . . . | 1 | | | | | | | | | | | 1 | | | | | | 1 |
| | 31 | 25 | 4 | 3 | 3 | 3 | 5 | 1 | 5 | 2 | 9 | 7 | 9 | 4 | 1 | | | 56 |

### 6. *Digestive Organs.*

| | Male. | Female. | Under 1 yr. | 1 to 2. | 2 to 5. | 5 to 10. | 10 to 15. | 15 to 20. | 20 to 30. | 30 to 40. | 40 to 50. | 50 to 60. | 60 to 70. | 70 to 80. | 80 to 90. | 90 to 100. | 100 to 110. | Total. |
|---|---|---|---|---|---|---|---|---|---|---|---|---|---|---|---|---|---|---|
| Abscess Abdominal . | | 1 | | | | | 1 | | | | | | | | | | | 1 |
| Aphthæ . . . | | 1 | 1 | | | | | | | | | | | | | | | 1 |
| Cancer of Bowels . | 1 | | | | | | | | | 1 | | | | | | | | 1 |
| " of Stomach . | 1 | 1 | | | | | | | | | 1 | | | 1 | | | | 2 |
| Cirrhosis of Liver . | 2 | | | | | | | | | 1 | 1 | | | | | | | 2 |
| Colic . . . . | 1 | | 1 | | | | | | | | | | | | | | | 1 |
| Congestion of Liver . | 1 | | 1 | | | | | | | | | | | | | | | 1 |
| Consumption of Bowels | 2 | 1 | 1 | | | | | | | 1 | | 1 | | | | | | 3 |
| Cramp of Stomach . | 1 | | | | | | | | | 1 | | | | | | | | 1 |
| Disease of Bowels . | 1 | 2 | 1 | | 1 | | | | | | 1 | | | | | | | 3 |
| " Liver . . | 4 | 2 | 1 | | 1 | | | | | 1 | 2 | | 1 | | | | | 6 |
| " Stomach . | | 1 | | | | | | | | | 1 | | | | | | | 1 |
| Dyspepsia . . . | 1 | | | | | | | | | | | | | 1 | | | | 1 |
| Dropsy Abdominal . | 1 | 3 | | | | | | | | 2 | 1 | 1 | | | | | | 4 |
| Gangrene of Bowels . | | 1 | | | | | | | | 1 | | | | | | | | 1 |
| Hemorrhage of Bowels. | 1 | 2 | | | | | | | | 1 | 1 | | 1 | | | | | 3 |
| " Stomach | | 2 | 1 | | | | | | | 1 | | | | | | | | 2 |
| Hernia . . . | | 1 | | | | | | | | | | | 1 | | | | | 1 |
| Ileus . . . . | 1 | | | | | | | | | 1 | | | | | | | | 1 |
| Inflammation of Bowels | 3 | 4 | 2 | 1 | 3 | | | | 1 | | | | | | | | | 7 |
| " Stom'h & Bow'ls | 25 | 27 | 9 | 4 | 5 | 5 | 2 | 2 | 8 | 7 | 2 | 3 | 5 | | | | | 52 |
| " Liver . . | 2 | 1 | | | | | | | | 1 | | 2 | | | | | | 3 |
| " Peritoneum . | 9 | 12 | 2 | | | 1 | | | 6 | 7 | 1 | 2 | | 1 | 1 | | | 21 |
| Intussusception . . | 1 | 1 | 2 | | | | | | | | | | | | | | | 2 |
| Jaundice . . . | 5 | 1 | 1 | | | | | | 3 | | | 1 | | 1 | | | | 6 |
| Obstruction of Bowels . | 1 | | 1 | | | | | | | | | | | | | | | 1 |
| Perforation of Bowels . | 2 | | | | | 1 | | | | | | 1 | | | | | | 2 |
| Scirrhus " . | 1 | | | | | | | | | | | 1 | | | | | | 1 |
| " of Stomach . | 2 | | | | | | | | | | 1 | 1 | | | | | | 2 |
| Strangulation of Bowels | 1 | 1 | | | | | | | | | | | | 2 | | | | 2 |
| Stricture " . | 1 | 2 | 1 | | | 1 | | | | | | 1 | | | | | | 3 |
| Teething . . . | 3 | 2 | 1 | 3 | | 1 | | | | | | | | | | | | 5 |
| Tumour of Abdomen . | | 1 | | | | | | | | | | 1 | | | | | | 1 |
| Ulceration of Stomach . | | 1 | | | | | | | | 1 | | | | | | | | 1 |
| Tympanites . . | 1 | | | | | | | 1 | | | | | | | | | | 1 |
| | 75 | 71 | 26 | 8 | 10 | 9 | 3 | 3 | 18 | 27 | 12 | 15 | 8 | 6 | 1 | | | 146 |

### 7. *The Urinary Organs.*

| | Male. | Female. | Under 1 yr. | 1 to 2. | 2 to 5. | 5 to 10. | 10 to 15. | 15 to 20. | 20 to 30. | 30 to 40. | 40 to 50. | 50 to 60. | 60 to 70. | 70 to 80. | 80 to 90. | 90 to 100. | 100 to 110. | Total. |
|---|---|---|---|---|---|---|---|---|---|---|---|---|---|---|---|---|---|---|
| Albuminuria . . | | 1 | | | | | | 1 | | | | | | | | | | 1 |
| Diabetes . . | 2 | 1 | | | | | | 1 | | | 2 | | | | | | | 3 |
| Disease of Bladder . | 1 | | | | | | | | | | | | | 1 | | | | 1 |
| " Kidneys . | 3 | 1 | | | | 1 | 1 | | | 1 | | | 1 | | | | | 4 |
| Inflam. of Bladder . | 1 | 1 | | | | | | | | | 1 | | 1 | | | | | 2 |
| " of Kidneys . | 2 | | 1 | | | | | | | | | 1 | | | | | | 2 |
| Retention of Urine . | 1 | | | | | | | | | | 1 | | | | | | | 1 |
| | 10 | 4 | 1 | | | 1 | 1 | 2 | | 1 | 4 | 1 | 2 | 1 | | | | 14 |

### 8. *Organs of Generation.*

| | Male. | Female. | Under 1 yr. | 1 to 2. | 2 to 5. | 5 to 10. | 10 to 15. | 15 to 20. | 20 to 30. | 30 to 40. | 40 to 50. | 50 to 60. | 60 to 70. | 70 to 80. | 80 to 90. | 90 to 100. | 100 to 110. | Total. |
|---|---|---|---|---|---|---|---|---|---|---|---|---|---|---|---|---|---|---|
| Child Bed . . . | | 4 | | | | | | | 2 | 2 | | | | | | | | 4 |
| Chlorosis . . . | | 1 | | | | | | | 1 | | | | | | | | | 1 |
| Disease of Uterus . | | 1 | | | | | | | | | | | | | 1 | | | 1 |
| Enlargemt. Prostate Gland | 2 | | | | | | | | | | | 1 | 1 | | | | | 2 |
| Hemorrhage from Uterus | | 4 | | | | | | | 1 | 1 | 2 | | | | | | | 4 |
| Inflammation " | | 4 | | | | | | 1 | 1 | 2 | | | | | | | | 4 |
| Ovarian Dropsy . | | 1 | | | | | | | | | 1 | | | | | | | 1 |
| Phlegmasia Dolens . | | 1 | | | | | | | | 1 | | | | | | | | 1 |
| Puerperal Fever . . | | 12 | | | | | | | 5 | 6 | 1 | | | | | | | 12 |
| " Mania . | | 2 | | | | | | 1 | | 1 | | | | | | | | 2 |
| Rupture of Uterus . | | 1 | | | | | | | | 1 | | | | | | | | 1 |
| Tumour " . . | | 1 | | | | | | | | | 1 | | | | | | | 1 |
| | 2 | 32 | | | | | | 2 | 10 | 14 | 5 | 1 | 1 | | 1 | | | 34 |

### 9. *Organs of Locomotion.*

| | Male. | Female. | Under 1 yr. | 1 to 2. | 2 to 5. | 5 to 10. | 10 to 15. | 15 to 20. | 20 to 30. | 30 to 40. | 40 to 50. | 50 to 60. | 60 to 70. | 70 to 80. | 80 to 90. | 90 to 100. | 100 to 110. | Total. |
|---|---|---|---|---|---|---|---|---|---|---|---|---|---|---|---|---|---|---|
| Abscess of Spine . | | 1 | | | | 1 | | | | | | | | | | | | 1 |
| Caries . . . | 1 | | | | | | | | 1 | | | | | | | | | 1 |
| Caries of Spine . . | | 1 | | | | | | | | 1 | | | | | | | | 1 |
| Disease " . . | 2 | 2 | | 1 | 1 | | | | 1 | | 1 | | | | | | | 4 |
| " of Hip . . | | 1 | | | | | | 1 | | | | | | | | | | 1 |
| Inflammation of Spine . | | 1 | | | | | | | | | | | | | 1 | | | 1 |
| Necrosis . . . | | 1 | | | | | | | | | | 1 | | | | | | 1 |
| Rheumatism . . | 5 | 3 | | | | 1 | 1 | | | 2 | 1 | 2 | | 1 | | | | 8 |
| Spinal Irritation . . | | 1 | | | | | | | 1 | | | | | | | | | 1 |
| | 8 | 11 | | 1 | 1 | 2 | 1 | 1 | 3 | 3 | 2 | 3 | | 1 | 1 | | | 19 |

10. *The Integumentary System.*

| | Male. | Female. | Under 1 yr. | 1 to 2. | 2 to 5. | 5 to 10. | 10 to 15. | 15 to 20. | 20 to 30. | 30 to 40. | 40 to 50. | 50 to 60. | 60 to 70. | 70 to 80. | 80 to 90. | 90 to 100. | 100 to 110. | Total. |
|---|---|---|---|---|---|---|---|---|---|---|---|---|---|---|---|---|---|---|
| Fistula . . . | | 1 | | | | | | | | 1 | | | | | | | | 1 |
| Ichthyosis . . . | 1 | | | 1 | | | | | | | | | | | | | | 1 |
| Purpura Hemorrhagica | 2 | 2 | 1 | | | | | 1 | | | | | | 1 | 1 | | | 4 |
| | 3 | 3 | 1 | 1 | | | | 1 | | 1 | | | | 1 | 1 | | | 6 |

11. *Old Age.*

| | Male. | Female. | Under 1 yr. | 1 to 2. | 2 to 5. | 5 to 10. | 10 to 15. | 15 to 20. | 20 to 30. | 30 to 40. | 40 to 50. | 50 to 60. | 60 to 70. | 70 to 80. | 80 to 90. | 90 to 100. | 100 to 110. | Total. |
|---|---|---|---|---|---|---|---|---|---|---|---|---|---|---|---|---|---|---|
| Old Age . . . | 15 | 24 | | | | | | | | | | 1 | 1 | 13 | 16 | 7 | 1 | 39 |

12. *From External Causes.*

| | Male. | Female. | Under 1 yr. | 1 to 2. | 2 to 5. | 5 to 10. | 10 to 15. | 15 to 20. | 20 to 30. | 30 to 40. | 40 to 50. | 50 to 60. | 60 to 70. | 70 to 80. | 80 to 90. | 90 to 100. | 100 to 110. | Total. |
|---|---|---|---|---|---|---|---|---|---|---|---|---|---|---|---|---|---|---|
| Asphyxia . . . | 10 | 6 | 15 | 1 | | | | | | | | | | | | | | 16 |
| Burns . . . | 4 | 5 | 1 | 3 | 4 | | | | 1 | | | | | | | | | 9 |
| Casualties . . . | 15 | 14 | 6 | 1 | | 3 | 1 | 1 | 4 | 8 | | | 4 | 1 | | | | 29 |
| Drowned . . . | 7 | | | | | | 1 | | | 4 | 1 | | 1 | | | | | 7 |
| Fracture of Leg . . | 3 | | | | | | | | 1 | 1 | 1 | | | | | | | 3 |
| " Skull . | 1 | 1 | | | | 1 | | | | | | 1 | | | | | | 2 |
| Intemperance . . | 5 | 2 | | | | | | | 3 | 1 | 1 | 1 | 1 | | | | | 7 |
| Poison . . . | 1 | 3 | | | | | | | 1 | 1 | 2 | | | | | | | 4 |
| Suicide . . . | 2 | 1 | | | | | | | 1 | 1 | | 1 | | | | | | 3 |
| Suffocation . . . | 6 | 1 | 2 | | | | | | | 5 | | | | | | | | 7 |
| | 54 | 33 | 24 | 5 | 4 | 4 | 2 | 1 | 11 | 21 | 5 | 3 | 6 | 1 | | | | 87 |
| Still Born . . . | 102 | 59 | 161 | | | | | | | | | | | | | | | 161 |
| Unknown . . . | 36 | 25 | 13 | 2 | 3 | 1 | 1 | 1 | 7 | 8 | 7 | 8 | 5 | 4 | 1 | | | 61 |

### TABLE NO. 3.

*Deaths for the First quarter of 1853, at fifteen distinct periods of life.*

| | |
|---|---|
| Under 1 year, . . . . . . | 470 |
| 1 to 2 . . . . . . . | 228 |
| 2 to 5 . . . . . . . | 338 |
| 5 to 10 . . . . . . . | 147 |
| 10 to 15 . . . . . . . | 37 |
| 15 to 20 . . . . . . . | 74 |
| 20 to 30 . . . . . . . | 254 |
| 30 to 40 . . . . . . . | 240 |
| 40 to 50 . . . . . . . | 149 |
| 50 to 60 . . . . . . . | 127 |
| 60 to 70 . . . . . . . | 112 |
| 70 to 80 . . . . . . . | 86 |
| 80 to 90 . . . . . . . | 42 |
| 90 to 100 . . . . . . . | 12 |
| 100 to 110 . . . . . . . | 1 |
| | 2317 |
| Still Born . . . . . . . | 161 |
| Total, | 2478 |

Included in the above Tables, were 201 from the Blockley Almshouse, 172 people of color, and 26 from the country; as follows:

| | Jan. | Feb. | March, | Total. |
|---|---|---|---|---|
| Almshouse, . . | 73 | 55 | 73 | 201 |
| Blacks, . . . . | 43 | 56 | 73 | 172 |
| Country, . . | 8 | 7 | 11 | 26 |
| | 124 | 181 | 157 | 399 |

